# Supplementary material for: Rationally designed mineralization for selective recovery of the rare earth elements
Source: Nat Commun. 2017 May 26;8:15670. doi: 10.1038/ncomms15670 (PMC5458567; doi:10.1038/ncomms15670)
Supplement: Supplementary Information — Supplementary Figures, Supplementary Tables and Supplementary Reference. [file ncomms15670-s1.pdf]

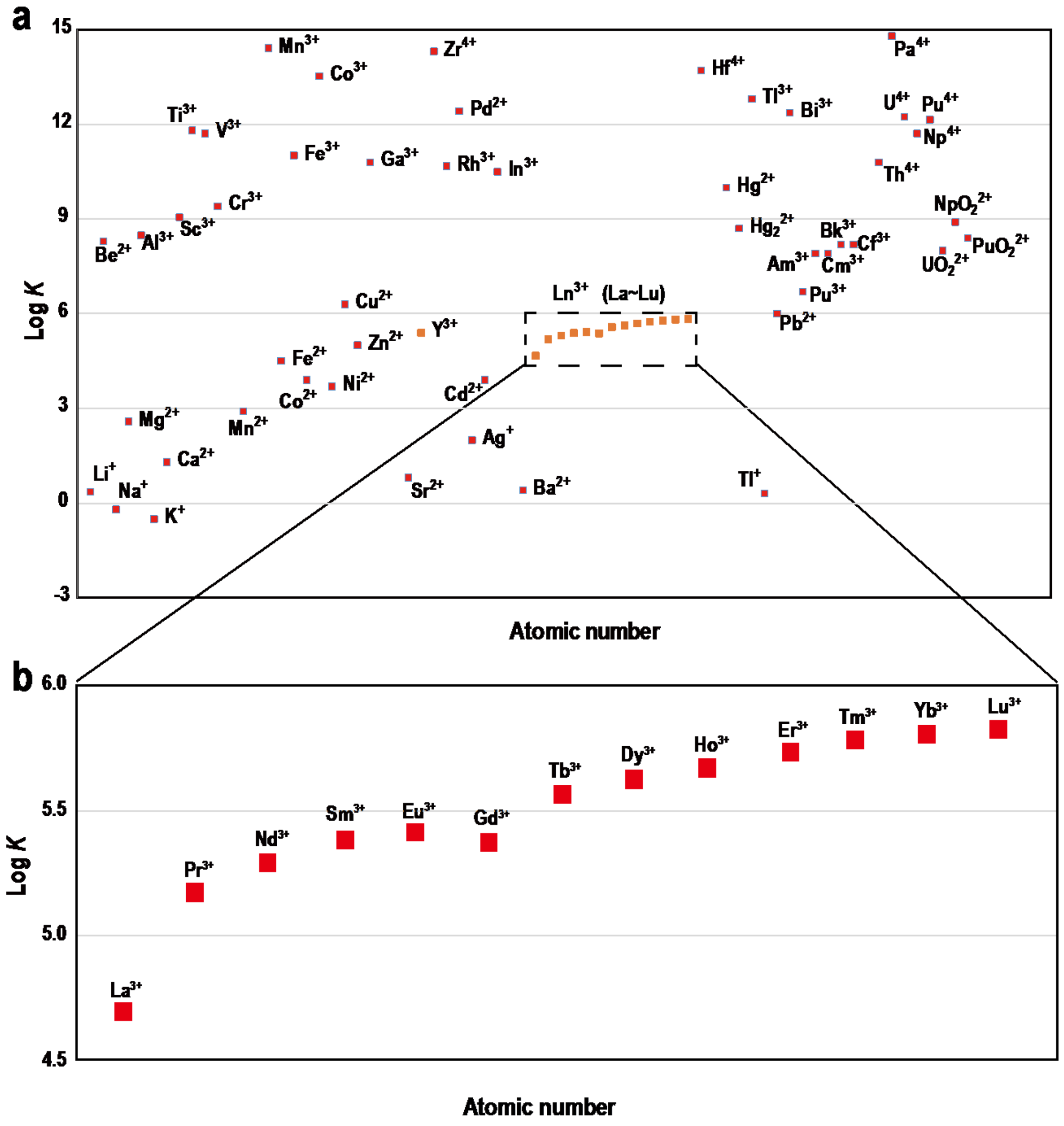

Supplementary Figure 1. Stability constants of metal monohydroxides. The log *K* values are summarized according to the atomic number of each element as determined in a previous study[1]. The log *K* value is the logarithm of the equilibrium constant for the following reaction (M indicates a metal): $M^{n+} + OH^- \rightleftarrows M(OH)^{n\text{-}1+}$ ($K = [M(OH)^{n\text{-}1+}]/[M^{n+}][OH^-]$). The atomic number of listed elements increases left to right.

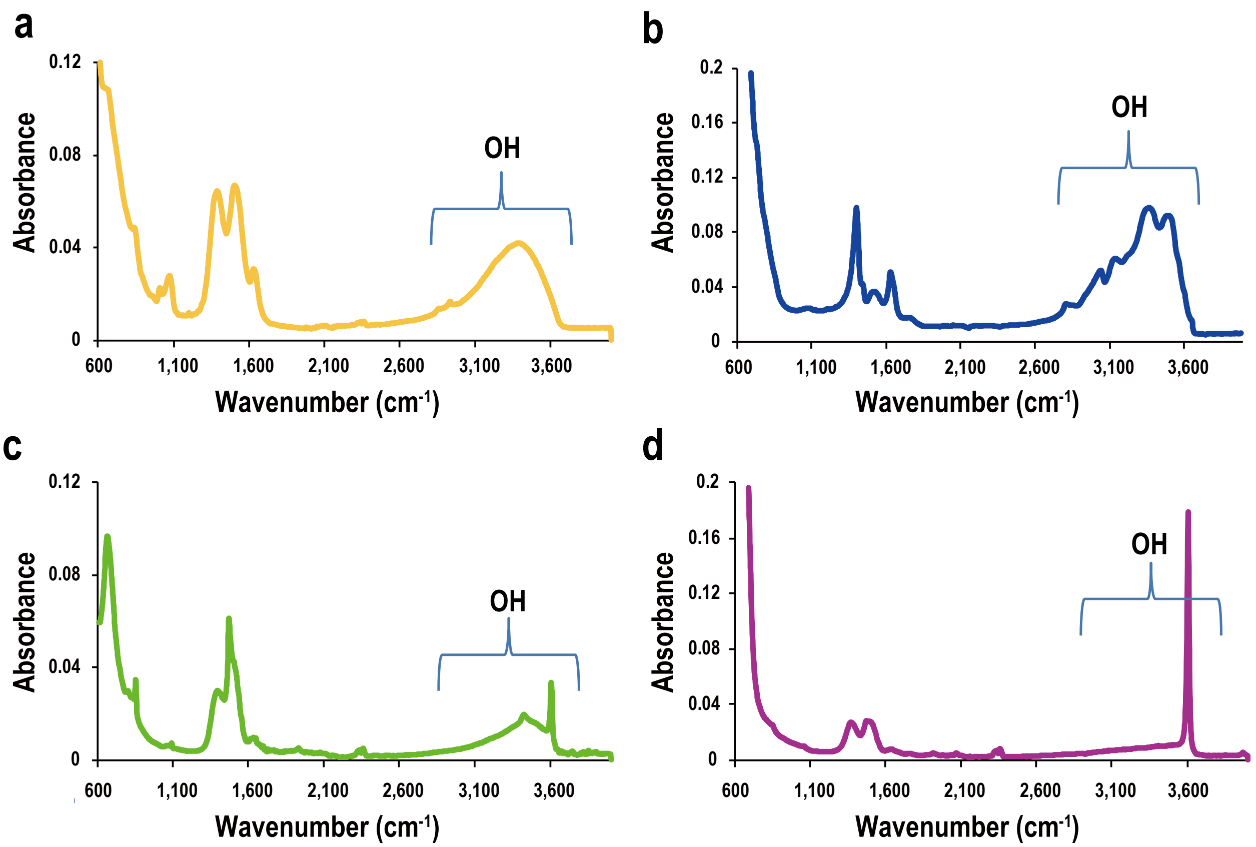

Supplementary Figure 2. FT-IR spectra of hydroxylated Ln_2O_3 nanoparticles. FT-IR spectra of **(a)** hydro-Dy_2O_3 and **(c)** hydro-Nd_2O_3 nanoparticles, which are used as the screening targets for biopanning in Supplementary Figure 3, and **(b)** $Dy(OH)_3$ and **(d)** $Nd(OH)_3$, which are used for the control measurements. The peaks observed at 2,800–3,600 cm^{-1} (bracket) indicate that hydroxyl groups are present on the particle surface.

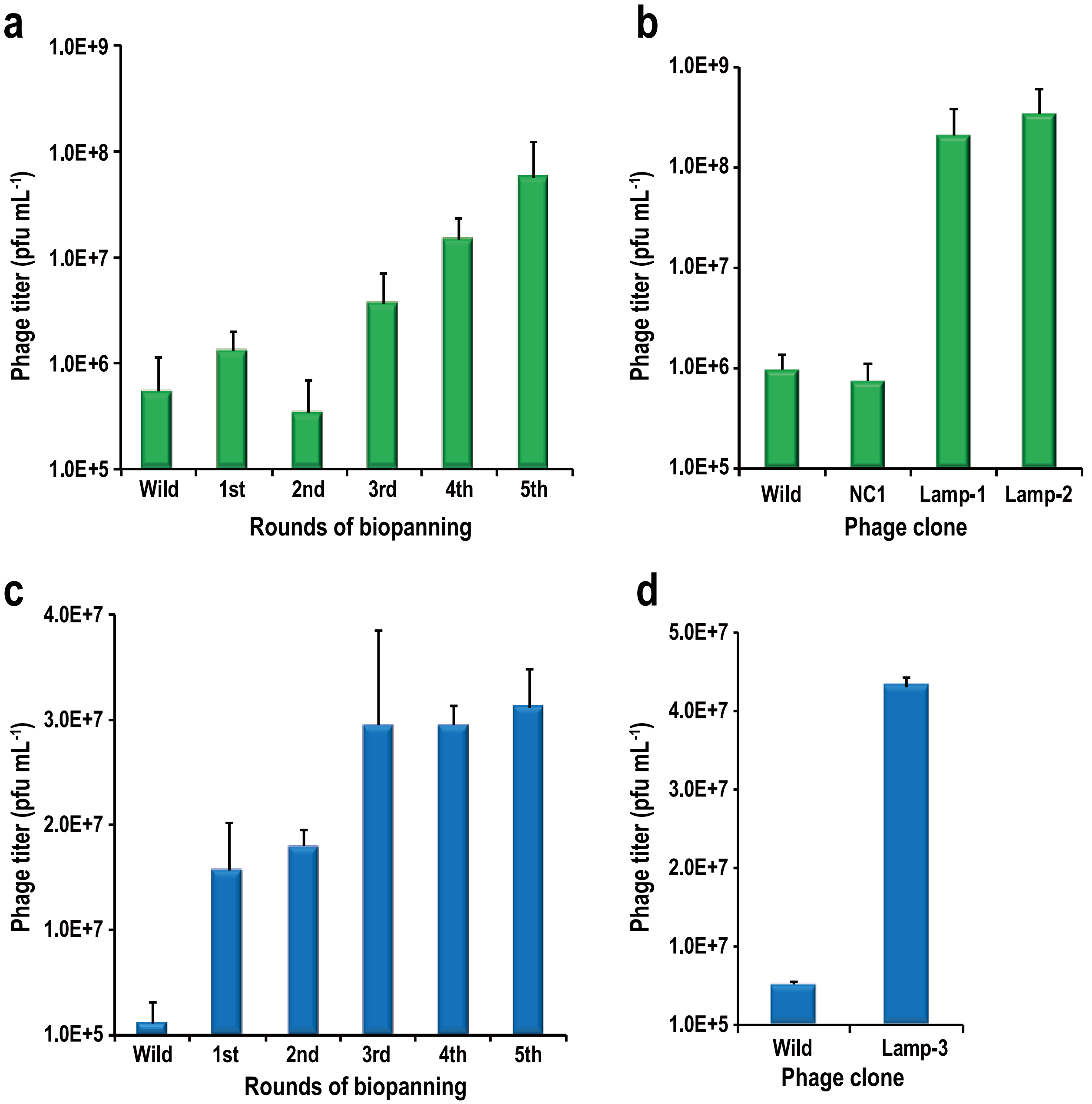

Supplementary Figure 3. Screening of hydro-Ln_2O_3 binding peptides from peptide libraries. The enrichment of **(a)** hydro-Dy_2O_3 and **(c)** hydro-Nd_2O_3 nanoparticle binding phages by five rounds of biopanning analysed by a titering assay. The binding ability of isolated single phages (NC1, Lamp-1, -2, and -3) with **(b)** hydro-Dy_2O_3 or **(d)** hydro-Nd_2O_3. The wild type phage was used as a control for all experiments. NC1 phage was isolated by screening against Dy_2O_3 and has little consensus with the Lamp sequence. Error bars represent the standard deviation of two experiments. The amino acid sequences of the selected clones are summarized in Supplementary Table 1.

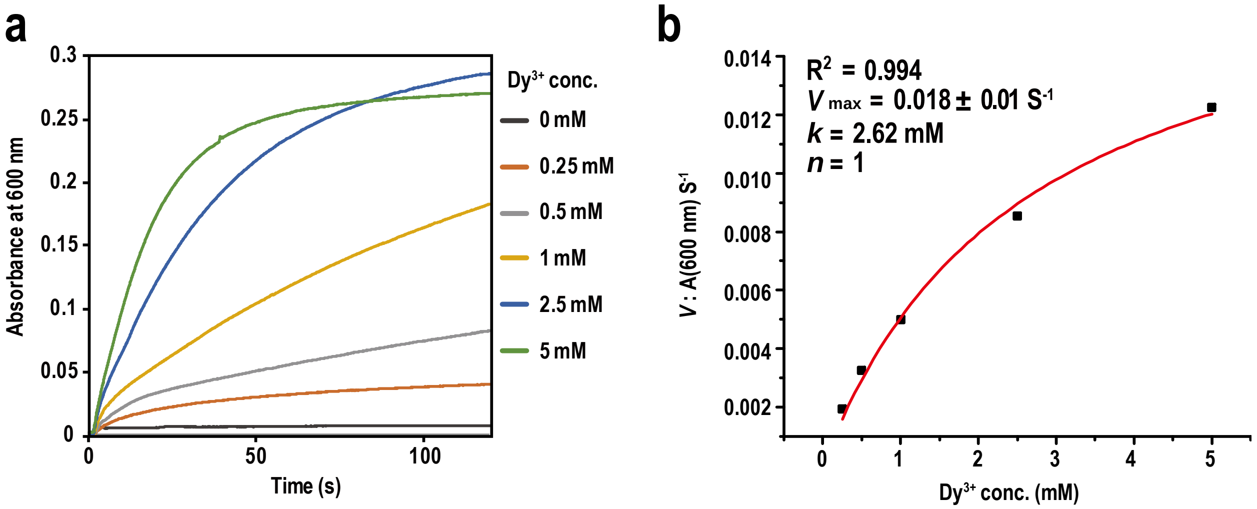

Supplementary Figure 4. Turbidity changes of the mineralization media. (a) The optical density of the mineralization media at 600 nm was recorded using a spectrophotometer. The Lamp-1 concentration was 100 µM in all experiments. Each curve represents the cumulative curve of three experiments. **(b)** The increasing speed of turbidity (V) at early stage (between 3 and 4 s after the measurement in **(a)**) is plotted as a function of Dy^{3+} concentration. The red line indicates the fitting curve analysed by the Hill equation: $V = V_{max} \cdot x/(k + x)$, where V_{max} is the maximum speed of increasing turbidity, and the k is half of the concentration at which the reaction speed reached V_{max}, and n is the stoichiometry (fixed to 1).

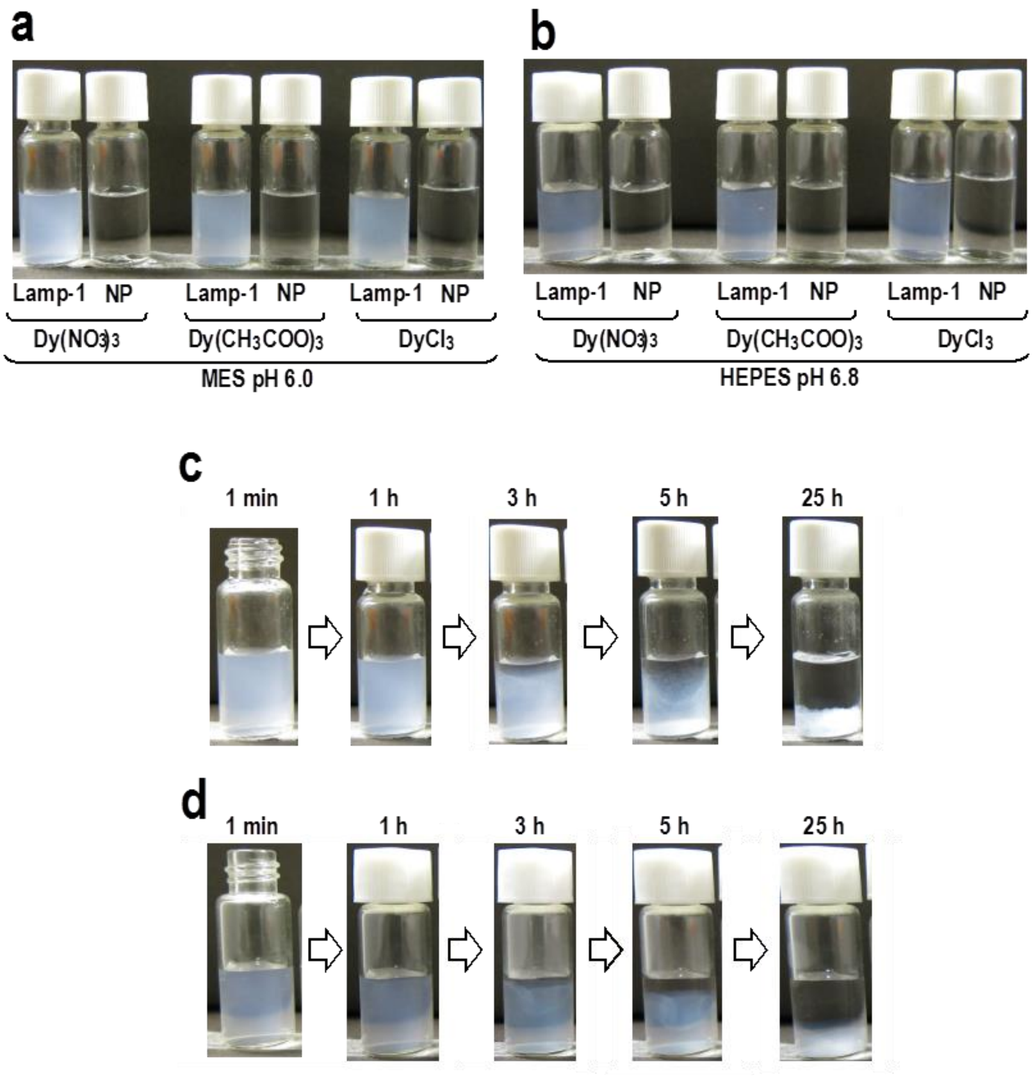

Supplementary Figure 5. Optical images of Dy^{3+} mineralization with Lamp-1 in various conditions. The Lamp-1 concentration was 100 µM in all experiments, and 50 mM of **(a)** MES or **(b)** HEPES buffer was used as the solvent. $Dy(NO_3)_3$, $Dy(CH_3COO)_3$, and $DyCl_3$ (3 mM) were used as the source of Dy^{3+}. **(c and d)** Optical images of Dy^{3+} mineralization at different reaction times. Lamp-1 (100 µM) with $Dy(NO_3)_3$ (3 mM) was incubated for 25 h in **(c)** MES (50 mM, pH 6.1) or **(d)** HEPES buffer (50 mM, pH 6.8).

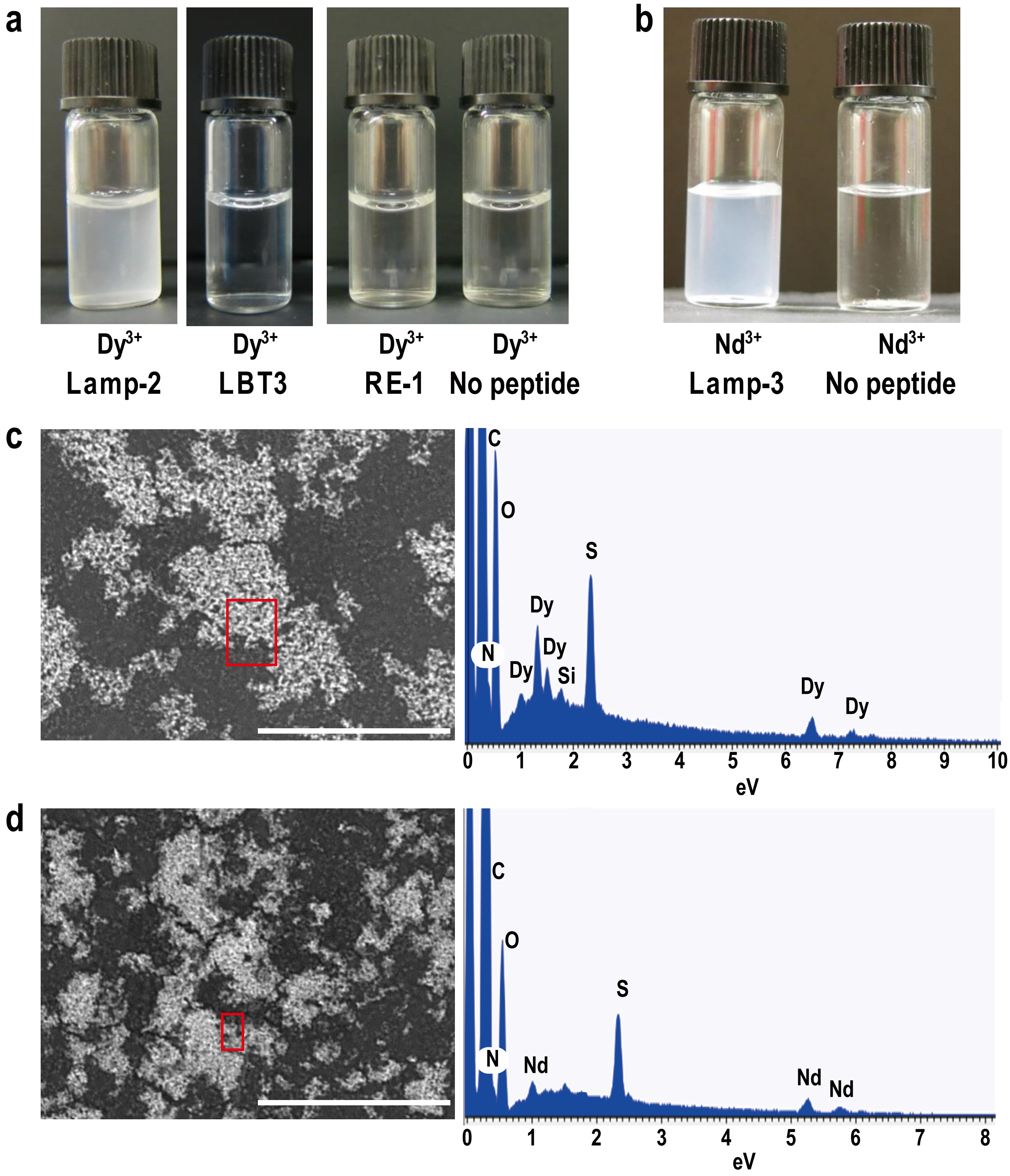

Supplementary Figure 6. Mineralization ability of Lamp-2 and Lamp-3. (a) Dy^{3+} or **(b)** Nd^{3+} was mixed with synthetic peptides in weak acidic buffer conditions (50 mM MES, pH 6.1). SEM (left) and EDX (right) images of the generated precipitate for **(c)** Lamp-2 and **(d)** Lamp-3. The red squares indicate the region for EDX analysis. Scale bars: 30 μm.

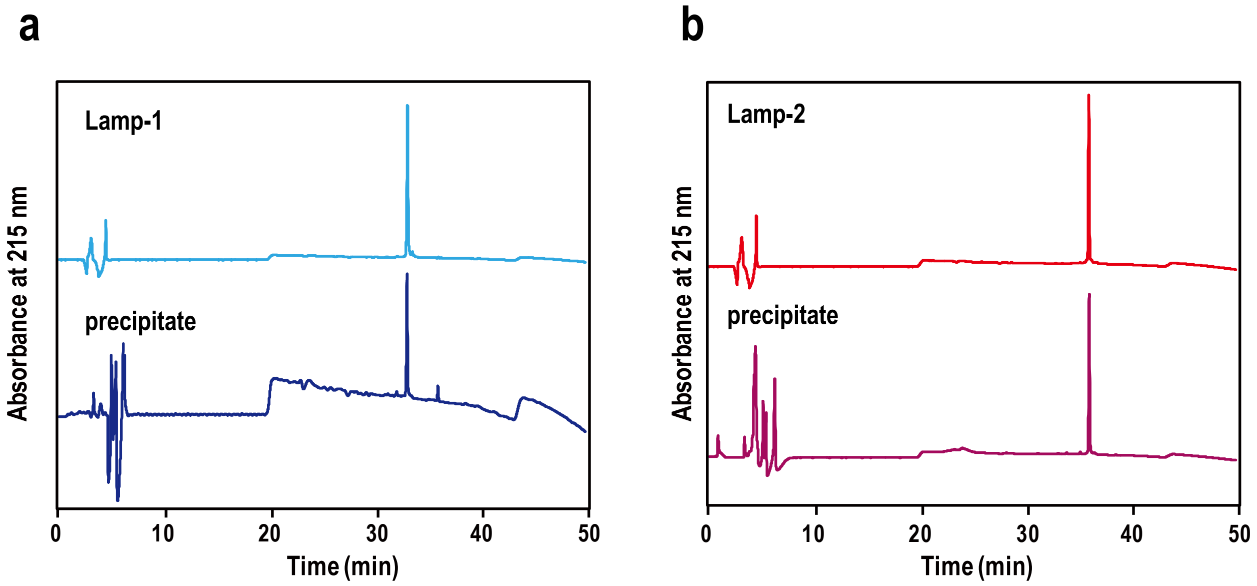

Supplementary Figure 7. The precipitates containing Lamp. The precipitated particles generated by the reaction between Dy^{3+} and **(a)** Lamp-1 or **(b)** Lamp-2 were dissolved in acidic solution (~pH 1.0) and analysed by RP-HPLC. The upper panels show the peptide only and the lower panels the dissolved precipitate.

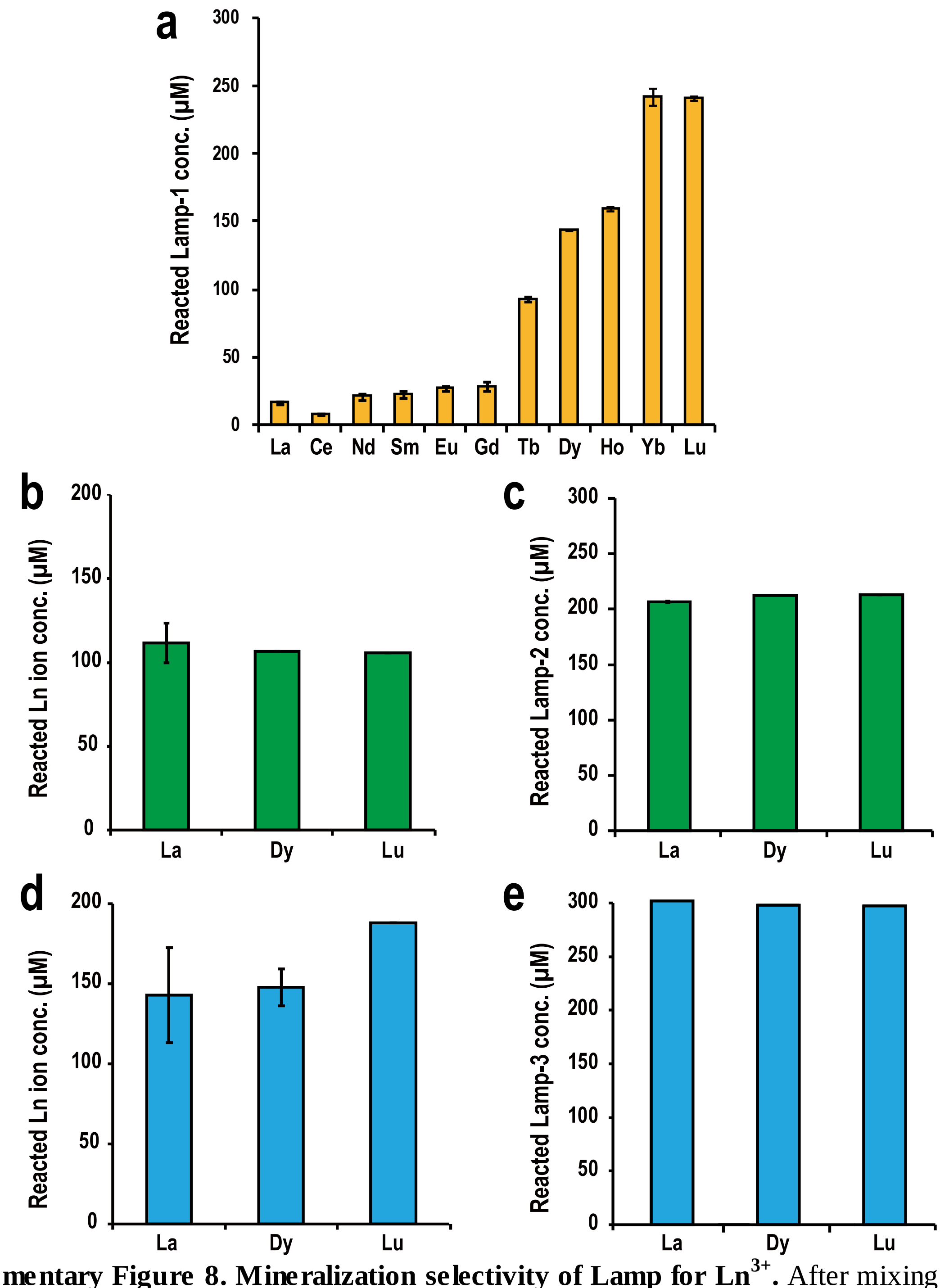

Supplementary Figure 8. Mineralization selectivity of Lamp for Ln^{3+}. After mixing 300 μM of **(a)** Lamp-1, **(b, c)** Lamp-2, and **(d, e)** Lamp-3 with Ln^{3+} (3 mM) at room temperature for 20 h, the generated particles were separated by centrifugation. The precipitated **(a, c, e)** peptides and **(b, d)** Ln^{3+} were determined by using a spectrophotometer and ICP-OES, respectively. All error bars represent the standard deviation of three experiments.

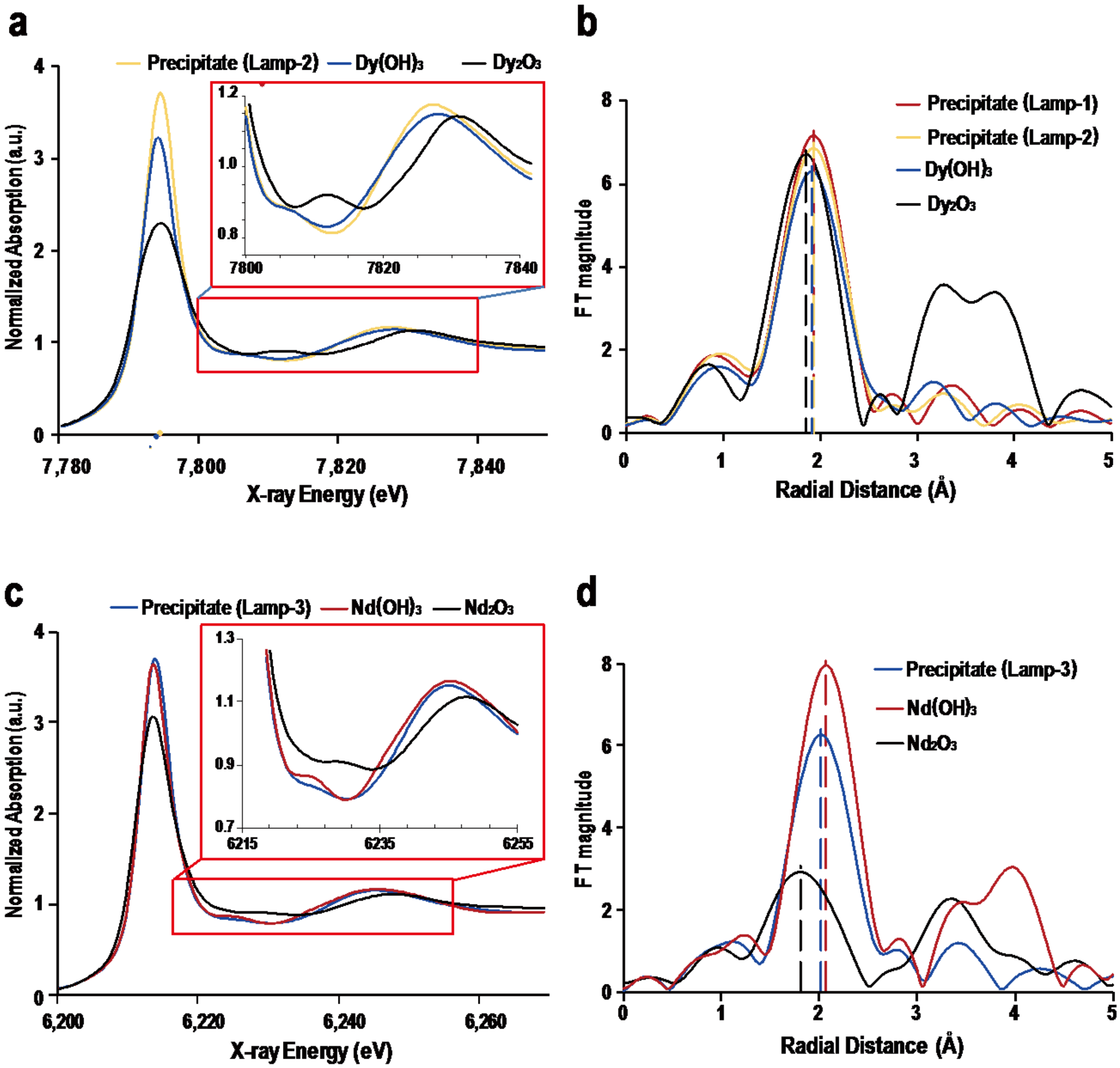

Supplementary Figure 9. XAFS measurements of generated precipitates. **(a)** Normalized Dy L_3-edge XANES spectra of the precipitate generated with Lamp-2 (yellow). The insert shows expanded spectra of the region in the red square. $Dy(OH)_3$ and Dy_2O_3 particles were used as a control (blue and black). **(b)** Fourier transform of the Dy L_3-edge EXAFS spectra of the precipitates with Lamp-1 (red) and Lamp-2 (yellow). The radial distance is not corrected for phase shifts. **(c)** Normalized Nd L_3-edge XANES spectra of the precipitate generated with Lamp-3 (blue). The insert shows the expanded spectra of the region in the red square. $Nd(OH)_3$ and Nd_2O_3 particles were used as a control (red and black). **(d)** Fourier transform of the Nd L_3-edge EXAFS spectra of the precipitates with Lamp-3.

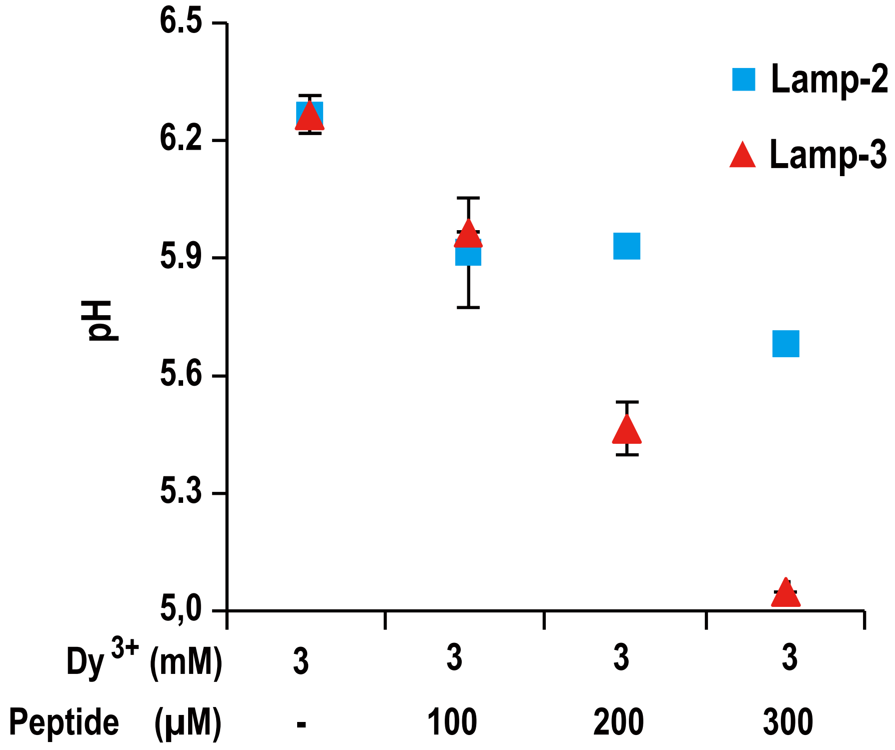

Supplementary Figure 10. Changes in the pH of the mineralization media containing Dy^{3+} at different peptide concentrations. Lamp and $Dy(NO_3)_3$ were each dissolved in 0.1 mM MES buffer and the pH was adjusted to 6.0–6.3. The pH value (vertical axis) was measured after mixing these two solutions. All error bars represent the standard deviation of two experiments.

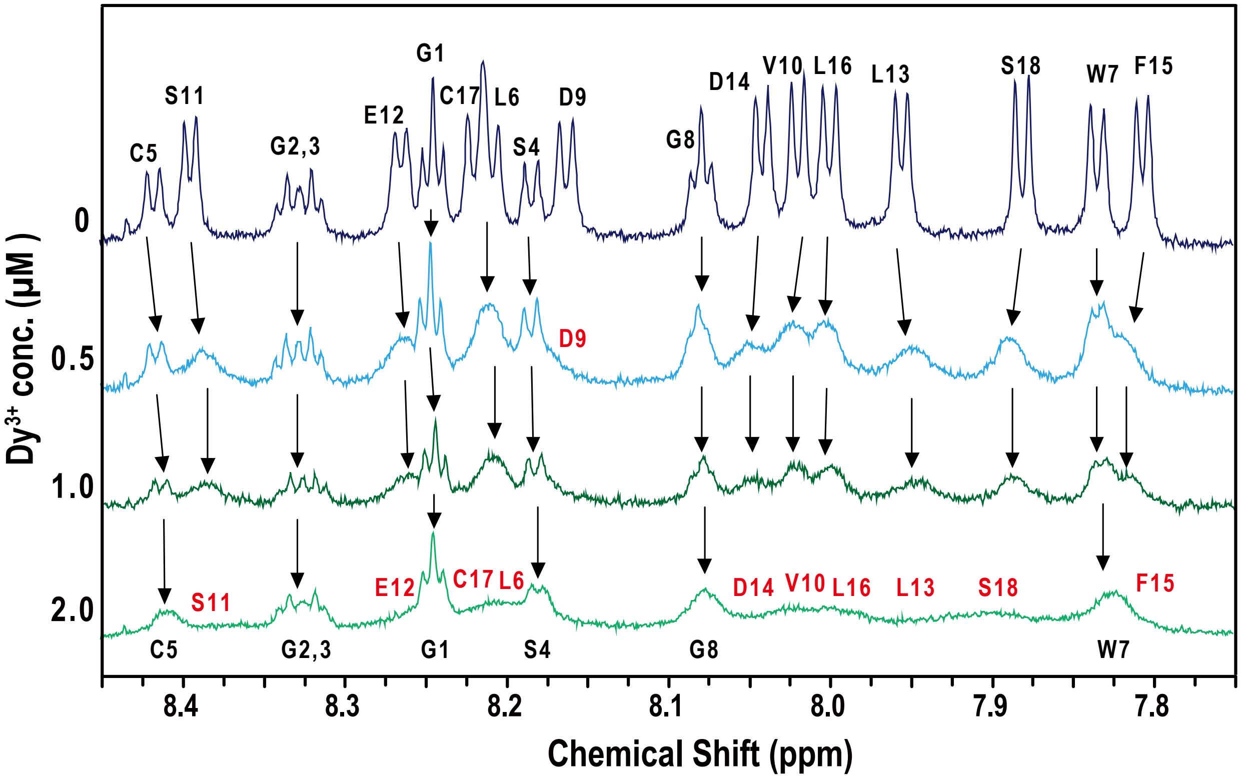

Supplementary Figure 11. NH chemical shift broadening of Lamp-1 with increasing amounts of Dy^{3+}. The assignments in black represent the residues that can be clearly recognized as a peak. The assignments in red show peaks largely broadened by the paramagnetic effect of Dy^{3+}.

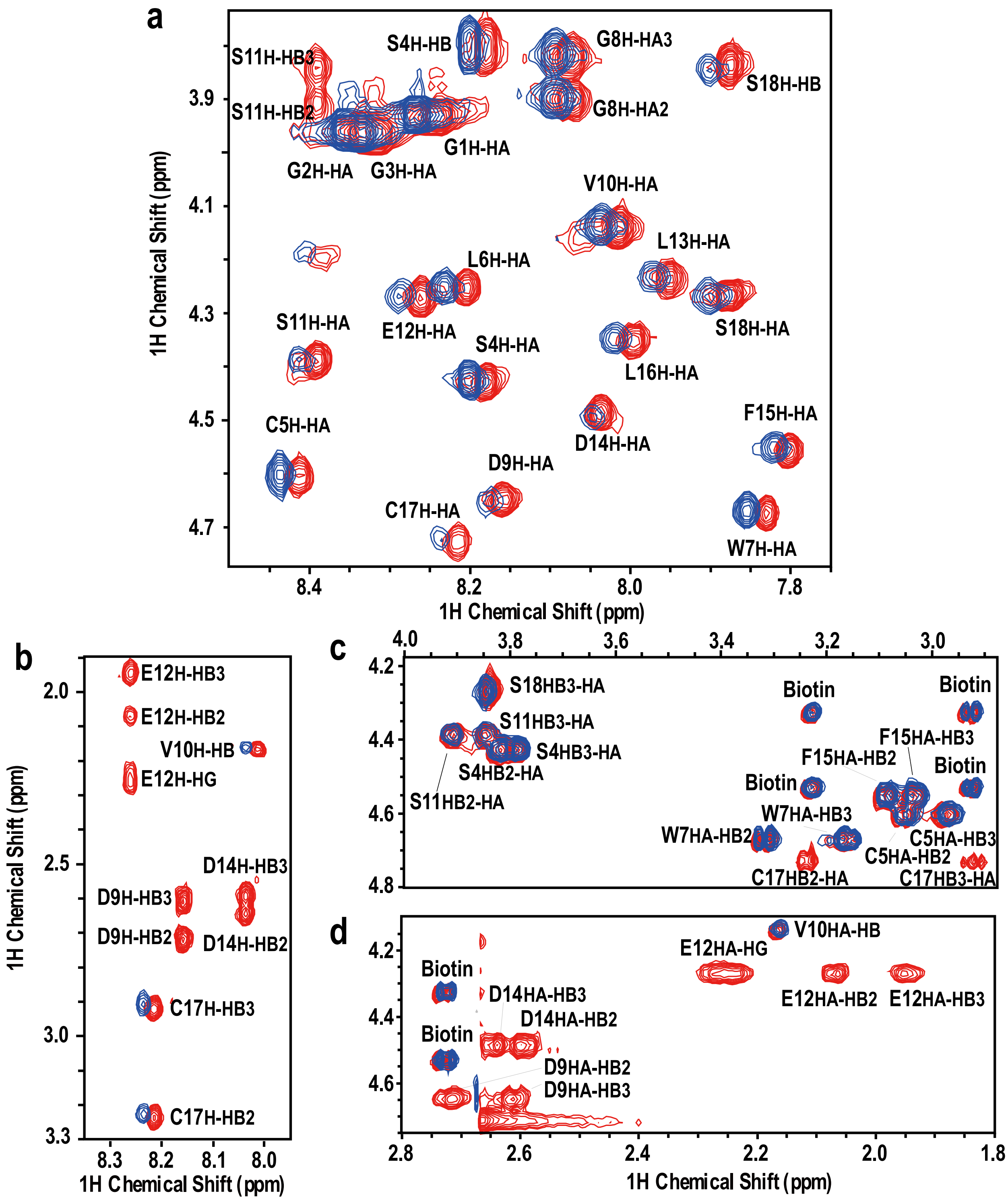

Supplementary Figure 12. Overlaid TOCSY spectra of Lamp-1 free (red) and bound to 0.5 μM Dy^{3+} (blue). Apparent spectral perturbations are observed in the **(a)** NH-Hα and **(b–d)** Hα-side chain regions. The cross peaks for the side chain of Asp9, Asp14, and Glu12 are severely affected, indicating that these residues contact Dy^{3+} first.

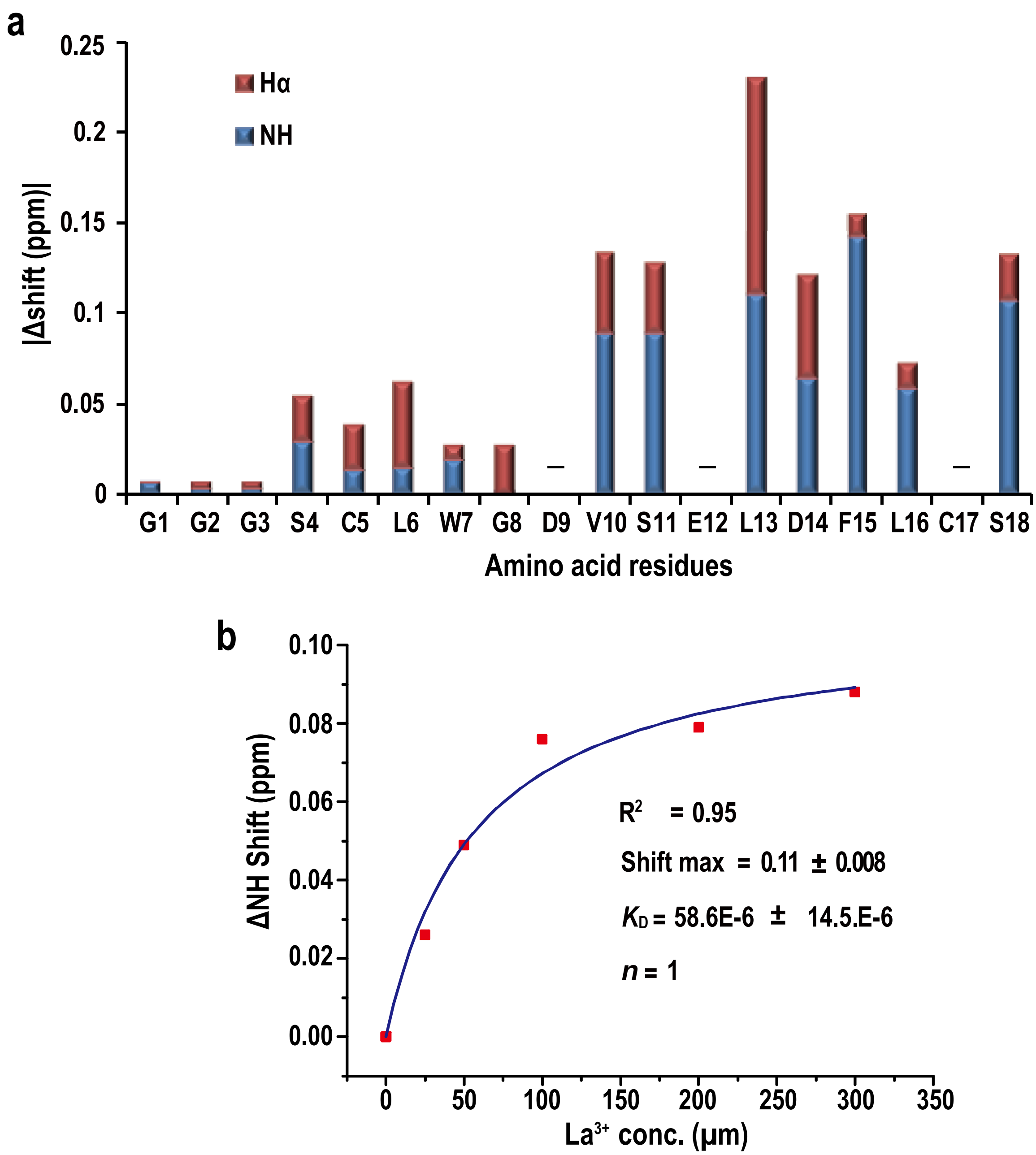

Supplementary Figure 13. The Lamp-1 peak shifts induced by La^{3+} titration. (a) The bars represent the absolute peak shift change (|Δshift|) calculated by subtracting the chemical shifts without La^{3+} from those following titration with 300 μM of La^{3+}. — represents residues that could not be assigned after titration with 300 μM of La^{3+}. **(b)** Calculation of the binding affinity (K_D) of Lamp-1 with La^{3+}. The NH shift of Val10 versus La^{3+} concentration was plotted and the curve was fitted to a one-site binding model: ΔNH shift = NH $\text{shift}_{max} \cdot x/(K_D + x)$.

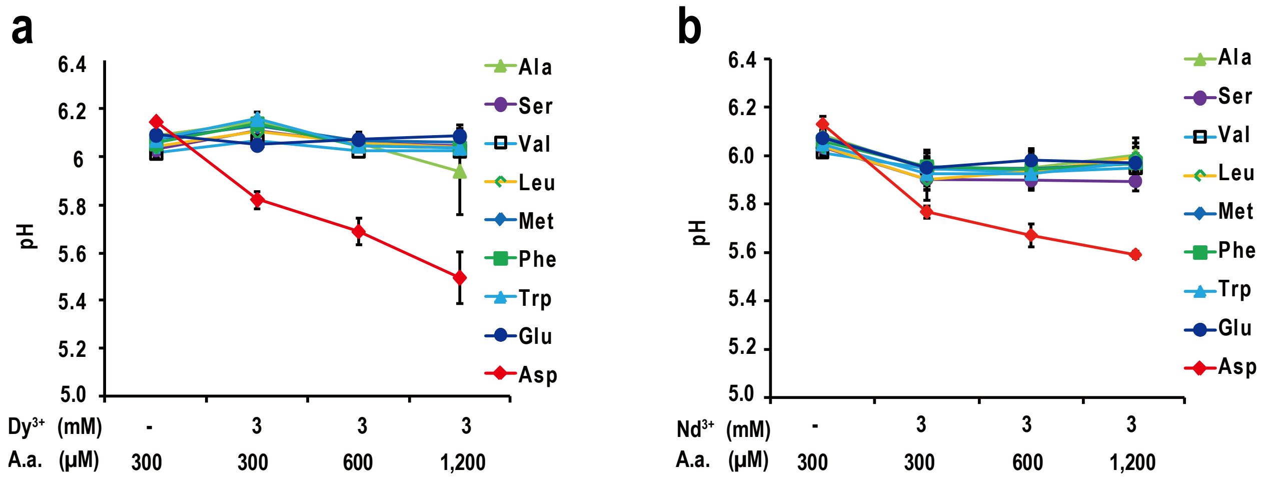

Supplementary Figure 14. Changes in the pH of the media containing Ln^{3+} at different amino acid concentrations. Each amino acid and $Ln(NO_3)_3$ were dissolved in 0.1 mM MES buffer and the pH was adjusted to 6.0–6.2. The pH value (vertical axis) was measured after mixing the amino acid solutions with (**a**) Dy^{3+} and (**b**) Nd^{3+}. All error bars represent the standard deviation ($n = 4$).

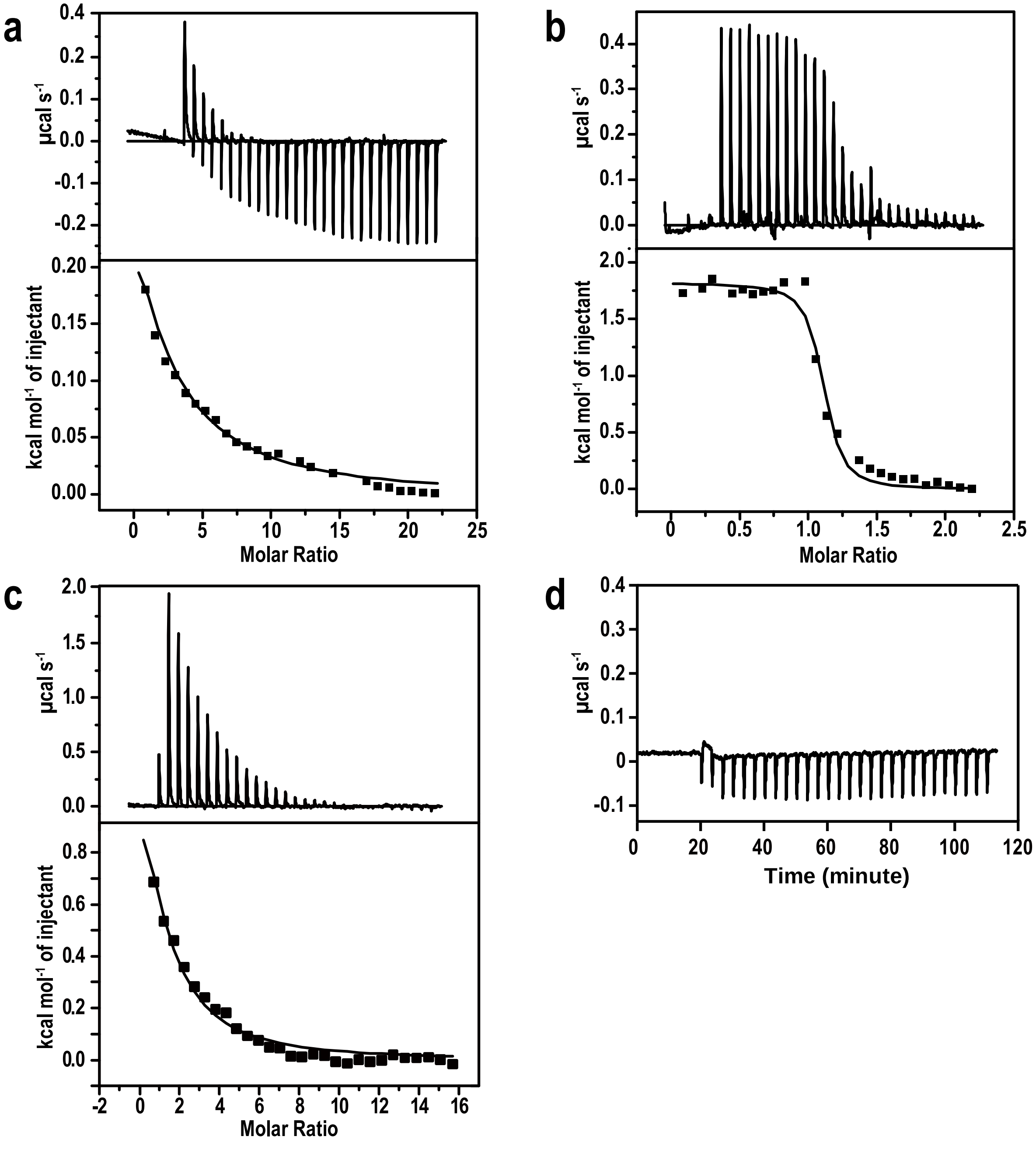

Supplementary Figure 15. Thermodynamic analysis of the mineralization reaction. ITC experiments for the reaction of Dy^{3+} with **(a)** Lamp-2 and **(b)** LBT3, and **(c)** Nd^{3+} with Lamp-3 in MES buffer. The upper panels show the calorimetric titration profile. The lower panels show a least squares fit of the data to the heat absorbed/mol of titrant versus the ratio of the total Dy^{3+} or Nd^{3+} concentration to the total peptide concentration. The solid line is the best fit of the data to a single binding site model using a non-linear least squares fit. The thermodynamic parameters are summarized in Supplementary Table 4. **(d)** Typical calorimetric titration profile of Dy^{3+} (5 mM) with MES buffer.

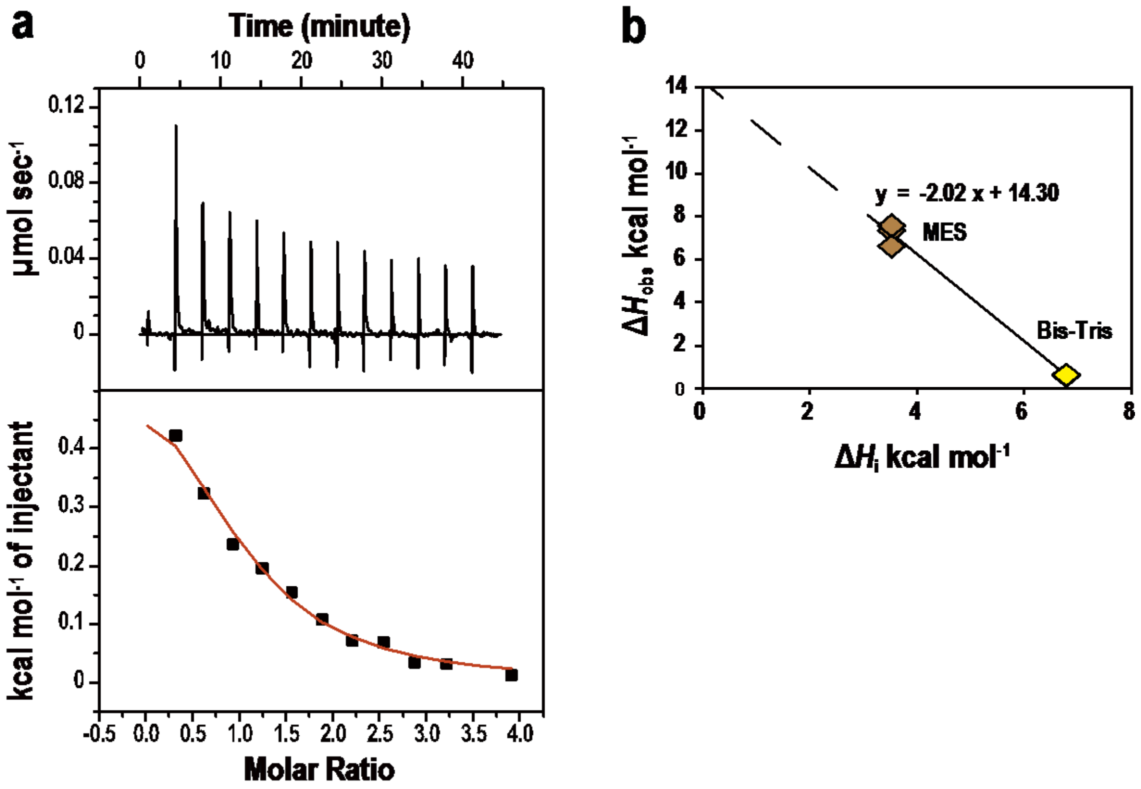

Supplementary Figure 16. Analysis of the protonation enthalpy for the reaction of Dy^{3+} with Lamp-1. (a) ITC experiments for the reaction of Dy^{3+} with Lamp-1 in Bis-Tris buffer. The upper panel shows the calorimetric titration profile. The lower panel shows a least squares fit of the data to the heat absorbed/mol of titrant versus the ratio of the total Dy^{3+} concentration to the total peptide concentration. The solid line is the best fit of the data to a single binding site model using a non-linear least squares fit. The thermodynamic parameters are summarized in Supplementary Table 4. **(b)** Plot of ΔH_{obs} (observed enthalpy change) versus ΔH_i (ionization enthalpy change) for the interaction of Dy^{3+} with Lamp-1 in MES or Bis-Tris buffer.

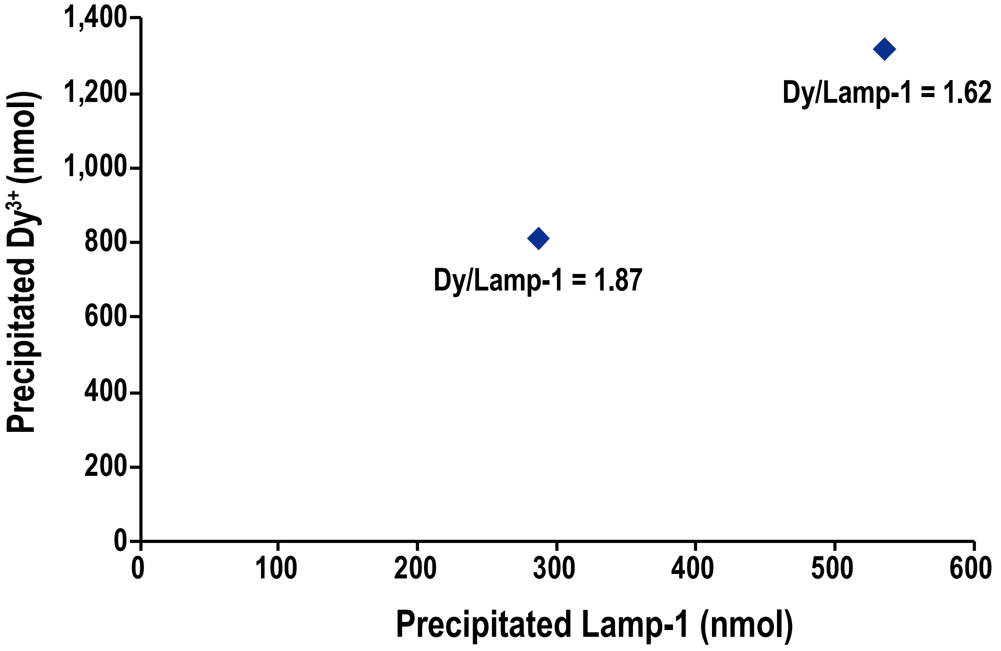

Supplementary Figure 17. Reaction stoichiometry of Dy^{3+} and Lamp-1 in synthetic seawater conditions. The amount of precipitated Dy^{3+} was plotted as a function of the amount of precipitated Lamp-1. The value displayed under each point indicates the reaction stoichiometry calculated using the following equation: precipitated Dy^{3+}/precipitated Lamp-1.

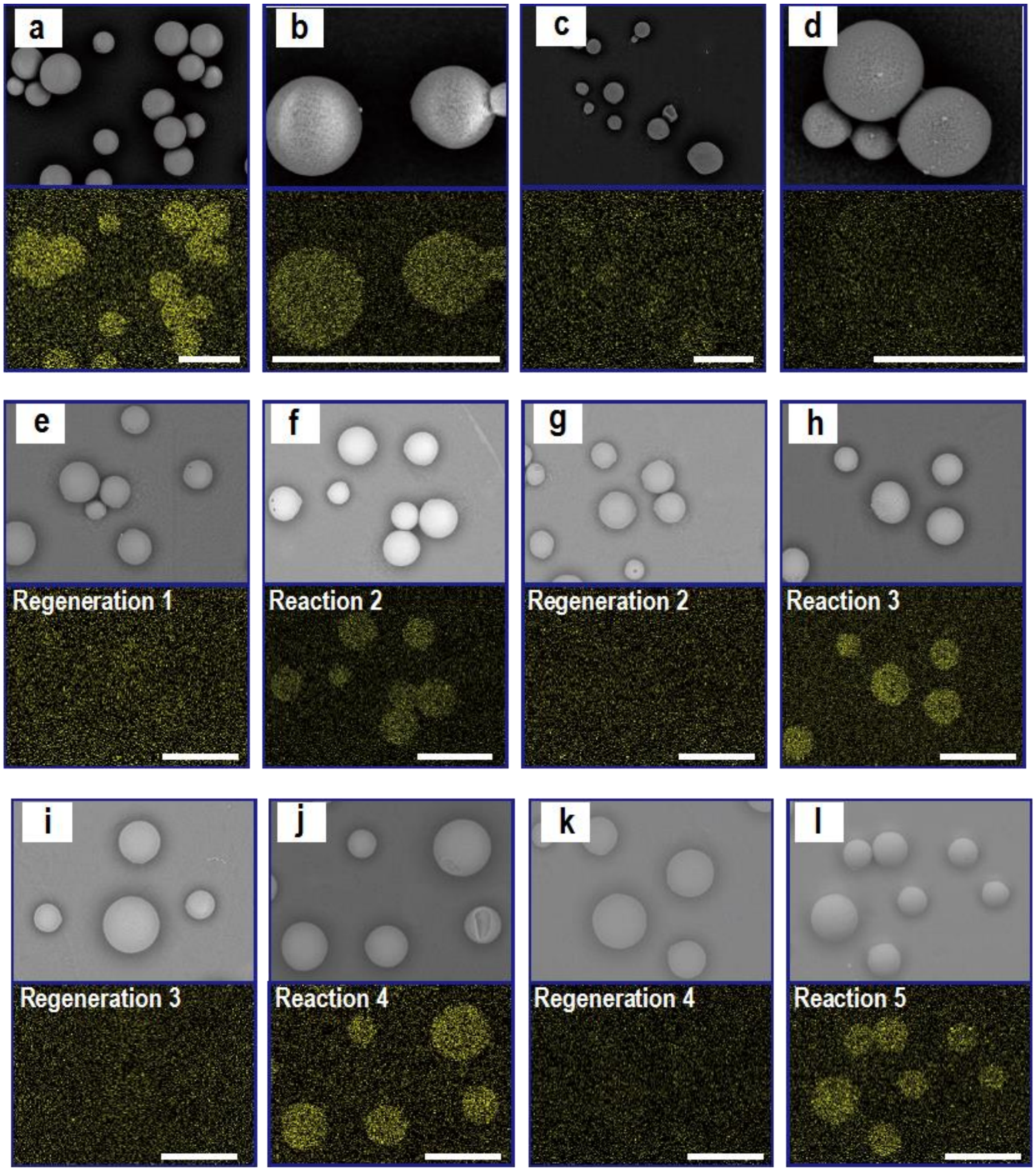

Supplementary Figure 18. Accumulation of Dy on the sepharose resin. SEM (upper panel) and EDX (lower panel) analyses of sepharose resins conjugated with **(a, b, e–h)** Lamp-1 and **(c and d)** control samples. **(e, g, i, and k)** Captured Dy was eluted with acetic buffer (50 mM) at pH 4.0, **(f, h, j, and l)** and the sepharose resin was recycled. Scale bars: 100 nm.

```
1                                             45                                            90
atgtcccctatactaggttattggaaaattaagggccttgtgcaacccactcgacttcttttggaatatcttgaagaaaaatatgaagag
1    M  S  P  I  L  G  Y  W  K  I  K  G  L  V  Q  P  T  R  L  L  L  E  Y  L  E  E  K  Y  E  E    30

91                                            135                                           180
catttgtatgagcgcgatgaaggtgataaatggcgaaacaaaaagtttgaattgggtttggagtttcccaatcttccttattatattgat
31   H  L  Y  E  R  D  E  G  D  K  W  R  N  K  K  F  E  L  G  L  E  F  P  N  L  P  Y  Y  I  D    60

181                                           225                                           270
ggtgatgttaaattaacacagtctatggccatcatacgttatatagctgacaagcacaacatgttgggtggttgtccaaaagagcgtgca
61   G  D  V  K  L  T  Q  S  M  A  I  I  R  Y  I  A  D  K  H  N  M  L  G  G  C  P  K  E  R  A    90

271                                           315                                           360
gagatttcaatgcttgaaggagcggttttggatattagatacggtgtttcgagaattgcatatagtaaagactttgaaactctcaaagtt
91   E  I  S  M  L  E  G  A  V  L  D  I  R  Y  G  V  S  R  I  A  Y  S  K  D  F  E  T  L  K  V    120

361                                           405                                           450
gatttcttagcaagctacctgaaatgctgaaaatgttcgaagatcgtttatgtcataaaacatatttaaatggtgatcatgtaacccat
121  D  F  L  S  K  L  P  E  M  L  K  M  F  E  D  R  L  C  H  K  T  Y  L  N  G  D  H  V  T  H    150

451                                           495                                           540
cctgacttcatgttgtatgacgctcttgatgttgttttatacatggacccaatgtgcctggatgcgttcccaaaattagtttgttttaaa
151  P  D  F  M  L  Y  D  A  L  D  V  V  L  Y  M  D  P  M  C  L  D  A  F  P  K  L  V  C  F  K    180

541                                           585                                           630
aaacgtattgaagctatcccacaaattgataagtacttgaaatccagcaagtatatagcatggcctttgcagggctggcaagccacgttt
181  K  R  I  E  A  I  P  Q  I  D  K  Y  L  K  S  S  K  Y  I  A  W  P  L  Q  G  W  Q  A  T  F    210

631                                           675                                           720
ggtggtggcgaccatcctccaaaatcggatggttcaactagttcaggtggaggttcgtgtttgtggggtgatgttagtgagctggatttt
211  G  G  G  D  H  P  P  K  S  D  G  S  T  S  S  G  G  G  S  C  L  W  G  D  V  S  E  L  D  F    240

721         732
ctgtgtagctga
241  L  C  S  *    244
```

Supplementary Figure 19. DNA (upper) and amino acid (lower) sequence of GST-Lamp-1. The underlined amino acid sequences show GST (blue) and Lamp-1 (green).

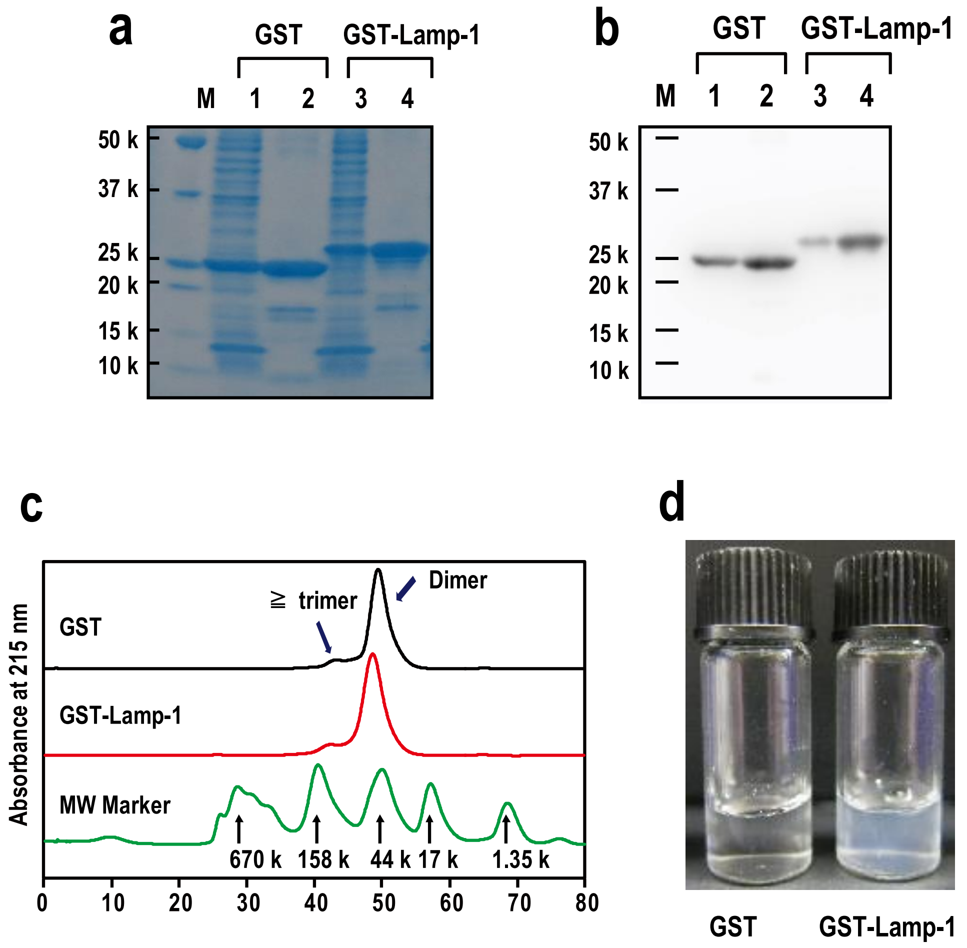

Supplementary Figure 20. The function of genetically engineered GST-Lamp-1. (a) SDS-PAGE and **(b)** Western blot analysis of the recombinant proteins before (lanes 1, 3) and after (lanes 2, 4) the purification. **(c)** The purified proteins were analysed by gel permeation chromatography using a Superdex200 10/300 column (GE Healthcare). **(d)** Optical image of Dy^{3+} mineralization by GST-Lamp-1 just after the reaction at room temperature. Each protein (10 µM) was incubated with $Dy(NO_3)_3$ (1 mM) in HEPES buffer (50 mM, pH 6.8) containing 150 mM NaCl.

Supplementary Table 1. The peptide library and isolated peptide sequences

Library Type	Diversity (pfu)	Concentration (pfu/ml)	Peptide	Sequence	Frequency
SCX_9CS	3.76E+07	8.11E+11	–	-	-
$SCX_{10}CS$	6.78E+06	9.56E+11	Lamp-2	SCLYPSWSDYAFCS	3/24
$SCX_{11}CS$	1.25E+07	5.00E+11	Lamp-1	SCLWGDVSELDFLCS	2/24
$SCX_{12}CS$	1.56E+06	9.00E+11	Lamp-3	SCPVWFSDVGDFMVCS	11/88

The T7 phage libraries displaying $SCX_{9–12}CS$ random peptides, where X represents the randomized amino acids, were constructed. T7 phage displays an average of 5–15 copies of the peptide on the phage surface. Two Cys residues cause the formation of an intra-disulfide bond.

Supplementary Table 2. Characteristics of the synthetic peptides

Peptide	Sequence	Length	pI	Other
Lamp-1	GGGSCLWGDVSELDFLCS	18 aa	3.38	cyclic
Lamp-2	GGGSCLYPSWSDYAFCS	17 aa	3.75	cyclic
Lamp-3	GGGSCPVWFSDVGDFMVCS	19 aa	3.49	cyclic
LBT3	GGGSFIDTNNDGWIEGDELLA	21 aa	3.2	linear
RE-1	GGGSACTARSPWICG	15 aa	8.23	cyclic
NC1	GGGSCVKGEFFRSISTCS	18 aa	8.23	cyclic

NC1: a peptide with little consensus with Lamp.
pI: isoelectric point.

Supplementary Table 3. Binding strength of synthetic peptides with hydroxylated Ln_2O_3

	EC_{50} (μM)	
	Hydroxylated Dy_2O_3	Hydroxylated Nd_2O_3
Lamp-1	0.1 ± 0.0	-
Lamp-2	1.2 ± 0.3	-
Lamp-3	-	0.6 ± 0.2
LBT3	17.5 ± 6.4	-
RE-1	24.2 ± 12.0	65.5 ± 18.5

N-terminally biotinylated peptides were used for detection.
The EC_{50} values were obtained from triplicate measurements.

Supplementary Table 4. Thermodynamic parameters of the peptide and Ln^{3+} reaction

Peptide	Target	ΔH (kcal/mol)	$-T\Delta S$ (kcal/mol)	ΔG (kcal/mol)	K ($x10^4 M^{-1}$)	N
Lamp-1[a]	Dy^{3+}	7.34 ± 0.05	-12.79 ± 0.06	-5.73 ± 0.01	1.59 ± 0.04	1[d]
Lamp-2[a]	Dy^{3+}	1.35 ± 0.04	-5.63 ± 0.06	-4.42 ± 0.06	0.17 ± 0.01	1[d]
LBT3[a]	Dy^{3+}	1.81 ± 0.04	-10.91 ± 0.27	-9.11 ± 0.27	477 ± 174	1.08 ± 0.01
RE-1[a]	Dy^{3+}	n.d	n.d	n.d	n.d	n.d
Lamp-3[a]	Nd^{3+}	2.41 ± 0.09	-7.48 ± 0.11	-5.08 ± 0.06	0.52 ± 0.05	1[d]
Lamp-1[b]	Dy^{3+}	0.52 ± 0.08	-6.92 ± 0.36	-6.12 ± 0.58	7.34 ± 2.52	1[d]
Lamp-1[c]	Dy^{3+}	14.41	20.14	-5.73	1.59	1[d]

ΔG was calculated using the equation $\Delta G = -RT \ln K$.
$-T\Delta S$ was calculated using the equation $\Delta G = \Delta H - T\Delta S$.
R: gas constant.
T: absolute temperature.
N: reaction stoichiometry.
n.d.: not detected (below the detection limit).
[a]MES buffer was used for pH regulation.
[b]Bis-Tris buffer was used for pH regulation.
[c]In consideration of the protonation enthalpy, the thermodynamic parameters were recalculated based on the data using MES buffer.
[d]N is assumed to be 1.

Supplementary References

1. Smith, M.R., Martell, E.A. & Eds. Critical Stability Constants Vol. 4 (Springer US, 1976).
